# Dignity Violations and Barriers to Dignity Assurance for Terminally Ill Patients at the End of Life: A Cross-Sectional Analysis

**DOI:** 10.3390/medicina58020294

**Published:** 2022-02-15

**Authors:** Eimantas Peičius, Gvidas Urbonas, W. David Harrison, Aušra Urbonienė, Jolanta Kuznecovienė, Rūta Butkevičienė, Kristina Astromskė, Ramunė Kalėdienė

**Affiliations:** 1 Department of Bioethics, Lithuanian University of Health Sciences, Tilžės St. 18, LT-47181 Kaunas, Lithuania; gvidas.urbonas@lsmuni.lt (G.U.); wdavidharrison@mac.com (W.D.H.); ausra.urboniene@lsmuni.lt (A.U.); jolanta.kuznecoviene@lsmuni.lt (J.K.); ruta.butkeviciene@lsmuni.lt (R.B.); 2 Department of Health Management, Lithuanian University of Health Sciences, Tilžės St. 18, LT-47181 Kaunas, Lithuania; kristina.astromske@lsmuni.lt (K.A.); ramune.kalediene@lsmuni.lt (R.K.)

**Keywords:** dignity, end of life, terminally ill, health professionals, Lithuania

## Abstract

*Background and Objectives*: Investigation into forms of behavior that violate dignity is not the typical way to look for means of dignity preservation, but it may be the optimal way to prevent improper behavior. Numerous studies document that maintaining and improving patient dignity at the end of life require an understanding of factors posing threats to dignity in health care organizations. This study aimed to assess associations between dignity-violating behaviors and barriers to the assurance of dignity in health care settings from the perspective of health professionals. *Materials and Methods*: An anonymous survey of health professionals was conducted in Lithuania in May 2021 by using a convenience sampling method (N = 168). Two scales were developed and included in the questionnaire. One scale measured respondents’ perceptions of Dignity Violations that they had witnessed. The other scale measured their opinions about Barriers to Dignity Assurance of terminally ill patients in clinical settings. Data analysis began with descriptive statistics, followed by exploratory principal component analysis (PCA) to identify the underlying structure of each scale. The variables assigned to distinct components in the PCA were combined into reflective latent variables in a path model. The path model of the relationships between the latent constructs was tested for significant links by implementing the partial least squares structural equation modeling technique. *Results*: Dehumanization, Humiliation, Inattentiveness, Control, Demonization, and Manipulation were identified as major forms of dignity-violating behavior. In addition, Organizational Barriers and Patient as an Obstacle were identified as two major types of barriers to the assurance of patient dignity. Both organizational and patient-oriented barriers were directly or indirectly associated with all forms of violations of patient dignity. *Conclusions*: The Dignity Violations scale showed potential for estimating professionals’ observations of dignity violations in health care settings. Perceived high workloads, staff shortages, insufficient resources, and lack of organizational support were identified as negative organizational factors that may result in increased risk of seeing patients as obstacles to providing care that preserves the dignity of terminally ill patients.

## 1. Introduction

The preservation of dignity is one of the most debated yet inconclusive challenges in health care. At the core of the framework of international bioethics, the notion of dignity entails the implementation of common professional values and ethical principles, including privacy, confidentiality, right to know, assurance of human rights, equal respectful relationships, and respect for patient autonomy [1]. In general, ethically appropriate care at the end-of-life should routinely promote the patient’s autonomy, involve the patient in shared decision-making, and be respectful of the values of the patient and his or her family [2].

The protection of human dignity is recognized as a sensitive and relevant issue “that might be of importance to people facing a life-limiting illness, and strategies to provide dignity-conserving care” in the context of the end of life are seen as essential [3]. Patients with severe and incurable or terminal illnesses may have limited or no opportunities to participate in daily and social life. They may lose their ability to act independently, making them dependent on the assistance of others. Consequently, they face a high risk of losing dignity. Moral and physical comfort, independence, privacy protection, interpersonal communication, hope, and many other characteristics are seen as important attributes of the concept of dying with dignity [4]. According to Chochinov, dignity-conserving practices include “Living in the moment”, “Maintaining normalcy”, “Finding spiritual comfort”, “Privacy Boundaries”, and others [5]. Hence, the imperative to protect the dignity of terminally ill patients presents ethical, legal and cultural challenges for health care providers worldwide. These challenges could be encompassed by the question of what are the right ways to treat the patient’s rights in the very specific context of the end-of-life.

However, some scholars have claimed that the notion of dignity lacks clarity and unified interpretation, and there are many conceptions and misconceptions of the meaning of dignity and its application in clinical practice [6]. Continuous research is necessary to understand ways to maintain dignity by identifying factors that are barriers to the goal of enhancing dignity. Dignity is also part of social life and “social dignity is usually understood in terms of countering its violation” [7]. The most vulnerable members of society may encounter existential inequalities, such as unequal distribution of autonomy and respect, limitations of freedom and communication with relatives, unequal distribution of resources, inequalities in income, and unequal distribution of health care access [8]. Dignity may be defined by its absence as well. Stoecker claimed that a “negative approach” to dignity is a more effective way to understand the essential attributes of dignity. He concluded that humiliation, degradation, and dehumanization are the three crucial starting points for an adequate understanding of human dignity [9].

Assessments of these inequalities can show how dignity is exercised or not in society. In addition, Jacobson has elaborated a taxonomy of dignity that includes elements of indignity, providing the “empirical base from which to develop strategies for enhancing human well-being” [10]. Therefore, Jacobson lists dignity-violating social processes that include the adverse interpersonal elements of rudeness, contempt, bullying, suspicion, body violation by intrusion, assault, and deprivation, among others. Moreover, as a member of society, a person might be degraded by exclusion, restriction, and discrimination, but greater humanity may be promoted through recognition, acceptance, and courtesy [10]. This approach has been used in several empirical studies focused on dignity-related challenges in the health care context [11,12,13].

Therefore, the question “what is dignity at the end of life” requires an assessment not only of its semantic meaning but also of how it is recognized in reality. We assume that, to improve dignity at end of life, first it is necessary to distinguish the meanings (indicators) of the violation of dignity from the experience of practitioners. This knowledge can be used in the contexts of health research, patients’ rights, and professional practice.

The aim of the current study was to assess the association between dignity-violating behavior and organizational and patient-related barriers to the assurance of dignity in health care settings.

## 2. Materials and Methods

The study implemented a cross-sectional survey design. The process began with a review and discussion of the relevant literature on the violations and barriers to dignity and how they might be related. Next was the development and administration of a questionnaire including scales of the observed violations of dignity and of the organizational and patient factors that might prove to be barriers to the assurance of dignity. Descriptive data were analyzed, scales were tested for validity, and specific components within each scale were identified. Then, a path model of the relations between the facets as latent variables was tested, and specific relationships were identified. Specifics of questionnaire development, sample characteristics, and data analysis are contained in this section.

### 2.1. Questionnaire Development

In order to ascertain professionals’ observations of the violations of dignity and factors that may be barriers to enhancing dignity, three team members drafted the questionnaire based on literature analysis and results of previous qualitative studies [14]. The remaining team members reviewed the draft and made changes until consensus was reached. The questionnaire consisted of two scales. The first scale (Dignity Violations) consisted of 24 statements developed to reflect the list of violations named by Jacobson [10]. Respondents were asked to mark on a 4-point Likert scale (“Never”, “Rarely”, “Frequently”, “Always”) if they had observed any of the listed “dignity violating” behaviors towards terminally ill patients in their professional settings. The second scale (Barriers to Dignity Assurance) was developed to identify potential barriers to assuring patient dignity. Eight contextual elements were identified as potential barriers for dignity assurance, based on scientific literature analysis, as well as based on the results of a qualitative study [14] conducted by the research team. The respondents were asked to mark on a 4-point Likert scale (“Never”, “Rarely”, “Frequently”, “Always”) the degree to which of the factors are or are not complicating dignity assurance for patients with incurable illnesses at the end of life.

### 2.2. Respondent Profile

The study was conducted in Lithuania in May 2021. During the study, electronic mail invitations were sent out to administrators of hospitals and hospices asking to share a link to the online survey with staff members who were working with terminally ill patients. A total of 168 persons responded. Ten questionnaires were not filled promptly and, therefore, were excluded from further data analysis. A total of 158 surveys were selected for data analysis.

The great majority (92.4%) of the respondents were women. Almost half the respondents (49.0%) reported that they had been working in this area for less than 10 years, 16.l% of respondents had been working for more than 30 years, others’ work experience was in between. Two-thirds of the respondents (63.9%) reported that they were working at a general hospital, 22.2% at a nursing hospital, 3.8% worked in an oncological hospital, and 3.2% were employed at a pension. The remaining 6.9% of professionals were working in other types of health care settings. Half of the respondents were nurses (52.5%), a quarter (24.7%) were physicians, 5.1% nurse assistants, and 17.7% reported that they possessed another type of qualification.

### 2.3. Data Analysis

Initially, the scales were tested for internal validity by computing Cronbach’s alpha coefficients. Next, principal component analysis (PCA) with varimax rotation method was performed separately for both scales to group variables to explore the underlying structure in both instruments separately. PCA and descriptive statistics were calculated using a statistical program IBM SPSS 27. The variables assigned to distinct components in the PCA stage later were combined into reflective latent variables in a path model. The hypothetical model of relationships between the latent constructs was tested for significant links by implementing the partial least squares structural equation modeling technique (PLS-SEM). All latent constructs were tested for convergent and discriminant validity. Convergent validity shows sufficient quality of latent constructs if factor loadings are above the value of 0.5 and the value of composite reliability for each latent variable is higher than 0.7 [15,16].

Discriminant validity was assessed to verify whether the constructs in a model are sufficiently distinct from each other. Discriminant validity was estimated by calculating the square root of the average variance extracted (AVE) and by comparing it with correlations between the latent constructs. Square roots of AVEs higher than correlations indicate sufficient discriminant validity [16]. In addition, heterotrait–monotrait ratios of correlations (HTMT) between all the pairs of the latent variables not exceeding the threshold of 0.85 (*p* < 0.05) supported sufficient discriminant validity of all of the facets [17]. The validity of the model of associations between the latent constructs of Barriers to Dignity Assurance and Dignity Violations was evaluated by calculating model fit and quality indices. In addition, the predictive validity for each endogenous variable, degree of vertical collinearity, and full collinearity variance was assessed. Direct and indirect coefficients of association were calculated together with their respective *p*-values, as well as Cohen’s f-squared effect sizes (f^2^) [18]. The statistical program WarpPLS version 7.0 was used for PLS-SEM analysis.

## 3. Results

Both the Dignity Violations scale (alpha = 0.93) and the scale of Barriers to Dignity Assurance (alpha = 0.85) showed sufficient reliability to be included for further analysis. Kaiser–Meyer–Olkin (KMO) coefficient values showed that the data of both Dignity Violations (KMO = 0.87) and Barriers to Dignity Assurance (KMO = 0.84) scales are appropriate for principal component analysis [19]. Six components with eigenvalues higher than 1 were extracted from the Dignity Violations scale (total variance = 67.3%), and two components were extracted from the Barriers to Dignity Assurance scale (total variance = 60.1%). The rotated component matrices of both scales are shown in Table 1.

**Figure 1:**
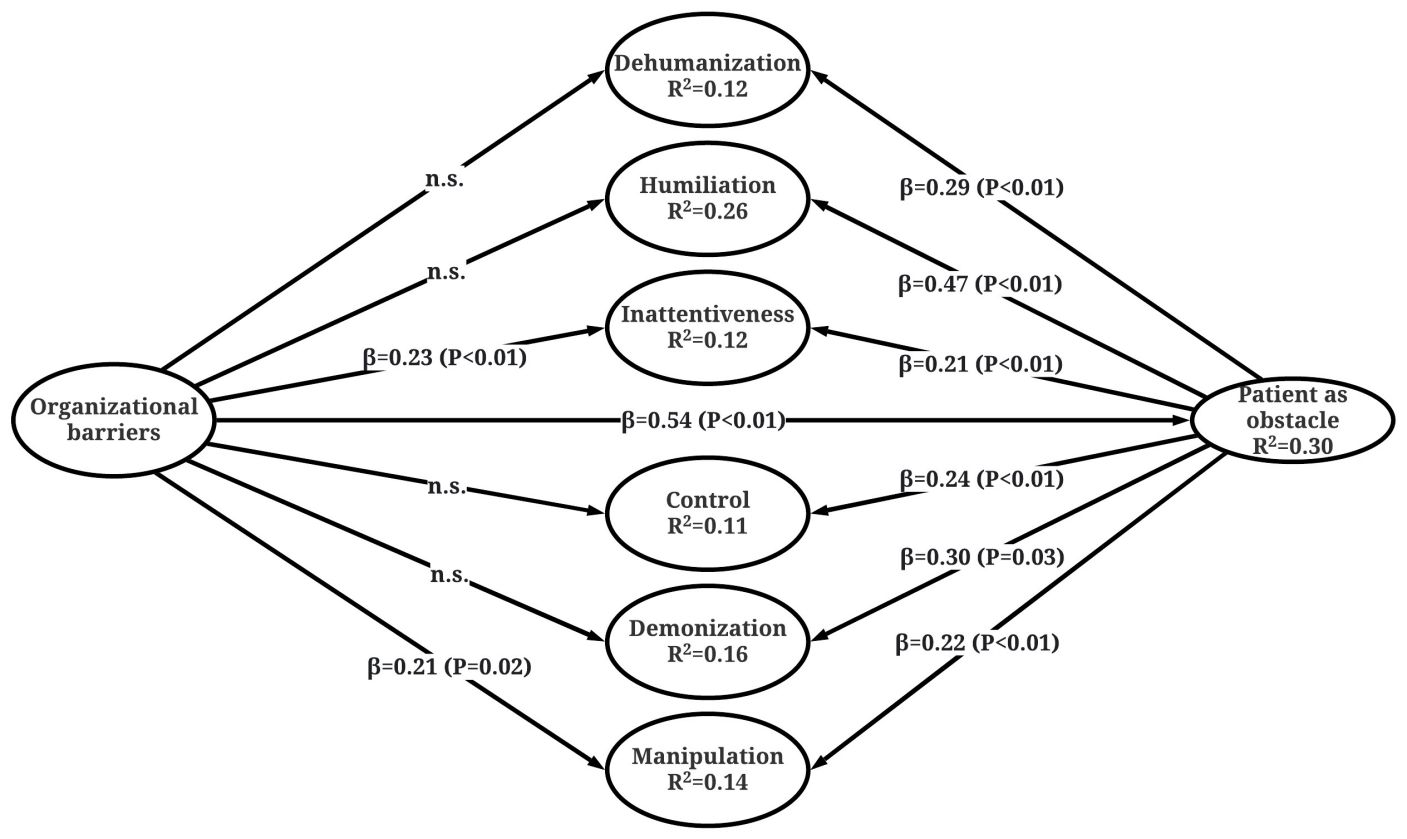
Path analysis of associations between the facets of Barriers to Dignity Assurance and Dignity Violations.

Furthermore, the items loaded on the same component in PCA were combined into latent variables for the PLS-SEM path model (Figure 1). Model fit indices (APC = 0.33, *p* < 0.001; ARS = 0.176, *p* < 0.03; AVIF = l.44; AFVIF = 2.07; SPR = 1.00, Tenenhaus (GoF) = 0.34, except for the Average adjusted R-squared coefficient (AARS = 0.169, *p* = 0.057) showed acceptable validity [18]. In addition, all the endogenous latent variables appeared as having sufficient.

Further, the items loaded on the same component in PCA were combined into latent variables for the PLS-SEM path model (Figure 1). Model fit indices (APC = 0.33, *p* < 0.001; ARS = 0.176, *p* < 0.03; AVIF = l.44; AFVIF = 2.07; SPR = 1.00, Tenenhaus (GoF) = 0.34, except for the Average adjusted R-squared coefficient (AARS = 0.169, *p* = 0.057) showed acceptable validity [18]. In addition, all the endogenous latent variables appeared as having sufficient predictive validity of the model with Q-squared coefficient values above zero [19]. The power analysis revealed that the sample size was sufficient to detect statistically significant 104 absolute path coefficients higher than 0.197 with the statistical power of 0.8.

The scores of composite reliability (CR) above the threshold of 0.7 value, the average variance extracted (AVE) coefficients above the 0.5 value, and indicator loadings above the threshold of 0.5 proved good convergent validity [18,20] of all the latent variables (Table 1). The heterotrait–monotrait ratios of correlations (HTMT) between all of the pairs of the latent variables (Table 2) not exceeding the threshold of 0.85 (*p* < 0.05) supported sufficient discriminant validity of all of the facets [17].

Since four of the six direct paths between Organizational Barriers and different facets of Dignity Violations were observed as insignificant (Figure 1), mediation analysis was initiated. The results revealed that the effects of Organizational Barriers on all the facets of Dignity Violations were fully or partially mediated via the variable of Patient as Obstacle (Table 3).

## 4. Discussion

This study attempted to outline potential issues and obstacles to dignified care by investigating the views and experiences of health professionals in clinical settings. Whereas the majority of research studies focused on the patients’ and carers’ perceptions of dignity, our study focused mainly on factors constituting breaches and potential violations of dignity as reported by health professionals. Jacobson’s taxonomy of dignity was proven to be a helpful tool to understand forms of dignity violation. Jacobson’s work contributes to the notion of “dignity screens” as a basis for improvement of the quality of services [10] and, importantly, to provide concrete suggestions for health care providers and decision-makers.

Systematic review and meta-analysis of studies related to the application of dignity and person-centered care for people with palliative care needs have highlighted several threats to dignity in acute hospital settings. These included pain and symptom management, communicative and culturally sensitive issues, assistance to patients and families in the process of death, and various ethical issues concerning disrespectful behavior towards patients [21]. Our study uncovered six such factors: Dehumanization, Humiliation, Inattentiveness, Control, Demonization and Manipulation. It is important to mention that the behaviors violating dignity were reported as appearing comparatively seldom. However, any behavior leading to dignity violations regardless of incidence rate is always serious to every vulnerable person, and zero tolerance strategies should be implemented by health care organizations.

In scientific literature, the term “dehumanization” refers to the denial, in part or whole, of the humanity of a person or group of persons [22]. It means that “technical and biological matters prevail over the human and the subjective” in contrast to the idea of establishing a form of accompaniment of the dying person, based on a humanist philosophy [23]. Dehumanizing, meaning not acknowledging a patient or making her or him feel inferior, treating him or her as an object violating his or her privacy or another aspect of care, and other negative aspects of the patient’s care were revealed to challenge dignity in daily practice. While safeguarding privacy is noted as one of the most important elements of dignity-conserving care [24], in our study, every second health professional reported having observed this violation of the patient’s privacy. Patient humiliation and inattentiveness by carers were other topics highlighted in our survey. Humiliation involved such medical staff behavior as forcing patients to act against their beliefs, making them feel unwelcome, mocking or putting physical or psychological pressure on them, and preventing them from satisfying their needs. Similarly, a cross-national Nordic study showed varieties of indignity in care, where deprivation of dignity due to psychological humiliation and the lack of privacy were also confirmed as main obstacles to dignified care in nursing homes [25].

In our study of health professionals’ perceptions, the category of Inattentiveness was associated with the disrespectful treatment of a patient, insufficient attention to diligence in care, degrading communication, and ignoring patients’ opinions or feelings in end-of-life care. Older adults face disrespectful attitudes in many cultures. It is recognized as “the most painful form of mistreatment identified by older adults”, especially in the developing countries [26]. Studies showed that ageism, stereotyping and negative prejudices against elderly patients still exist [27], and elders are frequently the subjects of abuse [28]. The aged might be treated in "a patronizing manner”, for instance, through the use of infantilizing language. Sometimes this occurs by “excluding them entirely from treatment conversations” [29] (p. 183) or simply ignoring their wishes [30]. Our study reveals that these dignity-violating features and such attitudes of health care providers were observed in end-of-life care in Lithuania. Disrespecting and ignoring patients’ opinions were identified as occasional issues by our sample. However, even the existence of such attitudes might seriously diminish patients’ dignity because of their vulnerability.

A vulnerable person can also become the object of demonization and manipulation. Our sample reported having witnessed dignity-threatening behavior that made patients feel dependent on the grace of others, as well as acts that limited patients’ control of their own lives. Health care providers’ attitudes that produce demonization, mistrust, and enmity against patients result in poor relationships and poor care [31]. Nearly every second health professional in the current study expressed their concerns about patients’ and their family carers’ unwillingness to collaborate and their unrealistic demands. These were identified as the most important factors that prevent them from enacting dignified care. Other studies have identified the importance of family non-acceptance of diagnoses and their unrealistic expectations and requirements [32]. Similarly, patients’ and families’ inadequate understanding of nursing care, their reluctance to participate in patient care, their anger, and their avoidance of the patient or medical staff have been identified as barriers to end-of-life care [33,34]. Perhaps sometimes there is a communication cycle whereby professionals label a patient or family as “difficult”, which leads to their negative interaction with the health professionals, as well as distancing attitudes and behavior on their part [34,35].

The study showed that health professionals identified distress resulting from excessive workload, lack of human resources, and absence of promotional opportunities at workplaces. A recent study also showed that health professionals expressed their concerns over staff shortages, meaning that patients were not attended to promptly [36]. They also identified insufficient resources and supplies that might have an important effect on interpersonal interactions (staff behavior). The quality of human and environmental resources can affect patient dignity [37]. The outcomes from a previous Lithuanian study reported that the lack of time for attending to patients’ and families’ spiritual and psychological needs in caring [33] for terminally ill patients might partially explain the factors of demonization and manipulation. Due to distress and other factors related to poor management, patients may be perceived as obstacles. The difficulties viewed by our sample as emerging in inpatient care might induce a feelings of disgust, resistance, and fatigue. These are conditions that could result in violations of dignity in end-of-life care. Several studies proved the importance of appropriate managerial interventions and support for staff members [36,37] as well as changes in organizational culture in terminally ill patient care settings. The aim of these organizational advances would be to provide time and resources to promote patient dignity properly [36,38].

The findings of this study confirm that specific facets of dignity violation hold significant implications for developing strategies to prevent behavior that violates dignity in the future. We assume that the prevention of dignity violation is a necessary, but not sufficient, condition for the enhancement of health care services in end-of-life care. The crucial next step is dignity promotion. For example, a recent Italian study demonstrated how dignity assurance in health care settings can be enhanced by providing privacy and adequate information, preserving patient autonomy and sense of control, addressing a staff’s decency, satisfying patients’ needs, even enhancing a staff’s sense of humor, as well as considering patients’ opinions [38]. Overall, we assume that understanding the barriers to dignity assurance can help decrease the largest obstacles and substantially increase the level of dignified end-of-life care.

### Strengths and Limitations

The main limitation of the study is the small sample size. Generalization should be done with caution because of the self-selection and self-reporting of participants. The results of the study should be seen as preliminary and do not prove causal links between the constructs. Despite the limitations, the study gives direction for more detailed SEM-based studies to identify organizational factors that provoke and that diminish violations of patient dignity in health care settings.

## 5. Conclusions

Our study revealed several results that are relevant for further discussion concerning strategies to improve dignity for terminally ill people in Lithuanian clinical settings. First, the Dignity Violations scale provided evidence that dignity violations in health care settings occur to some extent. Path analysis showed that dignity violations could arise from the perception that patients and families themselves could be obstacles to assuring patient dignity and that they were influenced by organizational problems, such as high workloads, staff shortage, insufficient resources, and lack of support from organizations.

Health care organizations should focus more on the elimination of organizational obstacles and the creation of dignity-supporting work environments that would improve the preservation of dignity of terminally ill patients and staff. 

## Figures and Tables

**Table 1:** Descriptive statistics, indicator loadings, and convergent validity.

Component	Indicators	IL *	Descriptive Statistics		
			Always/Frequently	Rarely	Never
**Dignity Violations**					
Dehumanization	CR * = 0.90; AVE * = 0.61; VIF * = 2.68				
	The patient was treated as if he/she was worth nothing	0.86	0.6%	19.1%	80.3%
	The patient was not acknowledged, as if he/she did not exist	0.84	1.3%	22.2%	76.6%
	The patient was made to feel inferior	0.81	2.5%	27.2%	70.3%
	The patient’s privacy was violated	0.69	12.0%	35.4%	52.5%
	The patient was viewed as a part of a group (e.g., ward) and not as a person	0.64	3.8%	25.9%	70.3%
Humiliation	CR = 0.88; AVE = 0.54; VIF = 2.97				
	The patient was prevented from satisfying basic needs	0.77	1.3%	15.9%	82.8%
	The patient was forced to act against his/her inner beliefs (e.g., a vegetarian is forced to eat meat)	0.72	1.3%	15.9%	87.3%
	The patient was viewed as disgusting, like a person with Hansen’s disease	0.77	1.3%	16.7%	82.1%
	The patient was labeled with derogatory terms	0.68	1.3%	13.3%	85.4%
	The patient was mocked	0.79	1.3%	9.0%	89.7%
	The patient was forced to feel unwelcome (e.g., in a resting room or the presence of others)	0.67	2.5%	18.5%	79.0%
Inattentiveness	CR = 0.86; AVE = 0.61; VIF = 1.83				
	The patient was treated disrespectfully	0.74	1.3%	36.1%	62.7%
	Communication with the patient was degrading (e.g., the patient was treated like a child)	0.84	8.9%	48.1%	43.0%
	The patient was not provided with enough attention, diligence, and/or care	0.78	7.0%	57.6%	35.4%
	A patient’s opinion and feelings were ignored	0.76	5.1%	46.5%	48.4%
Control	CR = 0.85; AVE = 0.74; VIF =1.91				
	The patient was made to feel dependent on the grace of others	0.86	6.3%	38.0%	55.7%
	The patient was granted limited control of his/her own life	0.86	8.9%	46.5%	44.6%
Demonization	CR = 0.87; AVE = 0.70; VIF = 2.12				
	The patient was viewed suspiciously as having bad intentions	0.80	0.6%	20.3%	79.1%
	The patient was demonized, depicted as a threat	0.87	1.9%	15.3%	82.8%
	Physical force or psychological pressure was used against the patient	0.84	1.3%	15.9%	82.8%
Manipulation	CR = 0.86; AVE = 0.67; VIF = 1.92				
	The patient was seen as a means to obtain a benefit (e.g., something of value)	0.90	1.3%	14.1%	84.6%
	The patients were treated differently according to their social status	0.78	9.6%	27.4%	63.1%
	The patient was lied to or manipulated for the benefit of others	0.77	1.3%	17.6%	81.0%
**Barriers to Dignity Assurance**					
Organizational barriers	CR = 0.87; AVE = 0.58; VIF = 1.50				
	Lack of human resources	0.76	51.9%	35.4%	12.7%
	Lack of support from management	0.73	28.0%	40.8%	31.2%
	Lack of means for care	0.75	23.4%	45.6%	31.0%
	Lack of motivation system	0.77	44.3%	30.4%	25.3%
Patient as obstacle	CR = 0.88; AVE = 0.72; VIF = 1.60				
	Patients unwilling to cooperate	0.83	21.2%	70.5%	8.3%
	Unrealistic expectations from patients	0.82	44.9%	49.4%	5.8%
	Family unwilling to cooperate	0.89	29.3%	59.2%	11.5%
	Unrealistic expectations from patients	0.82	44.9%	49.4%	5.8%
	Family unwilling to cooperate	0.89	29.3%	59.2%	11.5%

* Note: IL–indicator loading; CR–composite reliability; AVE–average variance extracted; VIF–full collinearity variance inflation factors.

**Table 2:** Discriminant validity.

	1	2	3	4	5	6	7	8
(1) Organizational barriers	**0.76**	0.51	0.25	0.30	0.34	0.27	0.30	0.32
(2) Patient as obstacle	0.63	**0.85**	0.32	0.43	0.32	0.32	0.26	0.33
(3) Dehumanization	0.31	0.39	**0.78**	0.65	0.61	0.62	0.53	0.60
(4) Humiliation	0.37	0.53	0.77	**0.74**	0.43	0.44	0.69	0.63
(5) Inattentiveness	0.43	0.41	0.74	0.54	**0.78**	0.55	0.33	0.41
(6) Control	0.37	0.45	0.83	0.60	0.77	**0.86**	0.46	0.46
(7) Demonization	0.37	0.32	0.64	0.85	0.43	0.65	**0.83**	0.48
(8) Manipulation	0.42	0.43	0.75	0.81	0.54	0.68	0.63	**0.82**

Note: Upper non-diagonal elements represent correlations; diagonal values represent square roots of average variances extracted; lower non-diagonal elements represent heterotrait–monotrait ratios of correlations.

**Table 3:** Mediation tests (Organizational Barriers -> Patient as Obstacle -> Dignity Violations).

	Indirect Effects	Total Effects	Type of Mediation
Dehumanization	0.19 (*p* < 0.001) f^2^ = 0.08	0.19 (*p* < 0.001) f^2^ = 0.05	Full
Humiliation	0.28 (*p* < 0.001) f^2^ = 0.05	0.28 (*p* < 0.001) f^2^ = 0.08	Full
Inattentiveness	0.11 (*p* < 0.01) f^2^ = 0.04	0.35 (*p* < 0.001) f^2^ = 0.12	Partial
Control	0.18 (*p* < 0.001) f^2^ = 0.05	0.18 (*p* < 0.001) f^2^ = 0.05	Full
Demonization	0.22 (*p* < 0.01) f^2^ = 0.07	0.22 (*p* < 0.01) f^2^ = 0.07	Full
Manipulation	0.12 (*p* < 0.05) f^2^ = 0.04	0.33 (*p* < 0.001) f^2^ = 0.11	Partial

## Data Availability

Datasets used and/or analyzed during the current study and the questionnaire in original language (Lithuanian) are available from the corresponding author on reasonable request.
